# Supplementary material for: The genetic architecture of the human thalamus and its overlap with ten common brain disorders
Source: Nat Commun. 2021 May 18;12:2909. doi: 10.1038/s41467-021-23175-z (PMC8131358; doi:10.1038/s41467-021-23175-z)
Supplement: Supplementary file 1 — Supplementary Information [file 41467_2021_23175_MOESM1_ESM.pdf]

Supplementary Information

for

The genetic architecture of the human thalamus and its overlap with ten common brain disorders

The Supplementary Information includes:

Supplementary Figs. 1-8 on pages 1-15

Supplementary Figures

a Whole thalamus-associated locus at chromosome 7

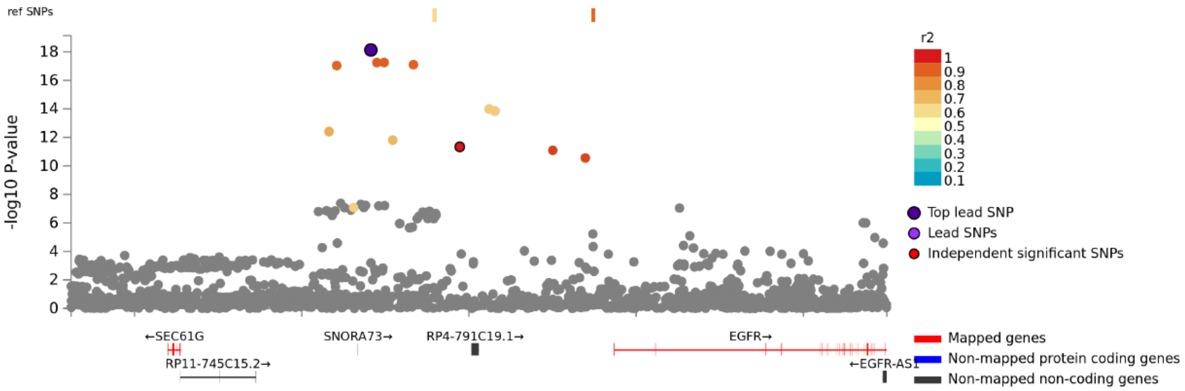

b Anterior nuclei-associated locus at chromosome 4

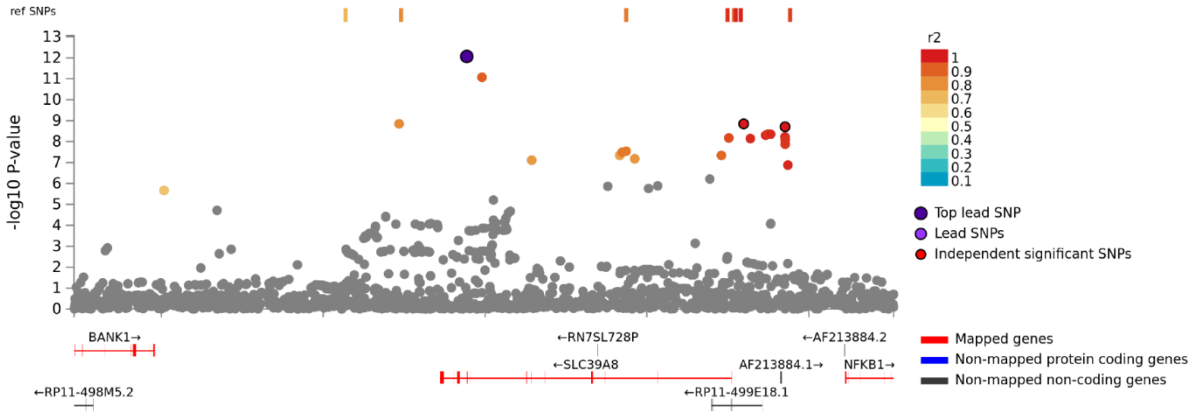

c Lateral nuclei-associated locus at chromosome 11

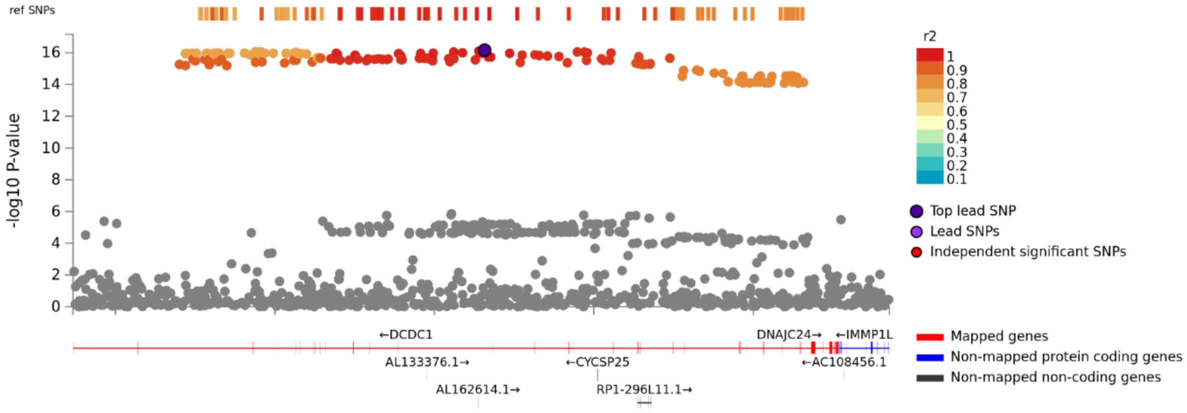

d Ventral nuclei-associated locus at chromosome 3

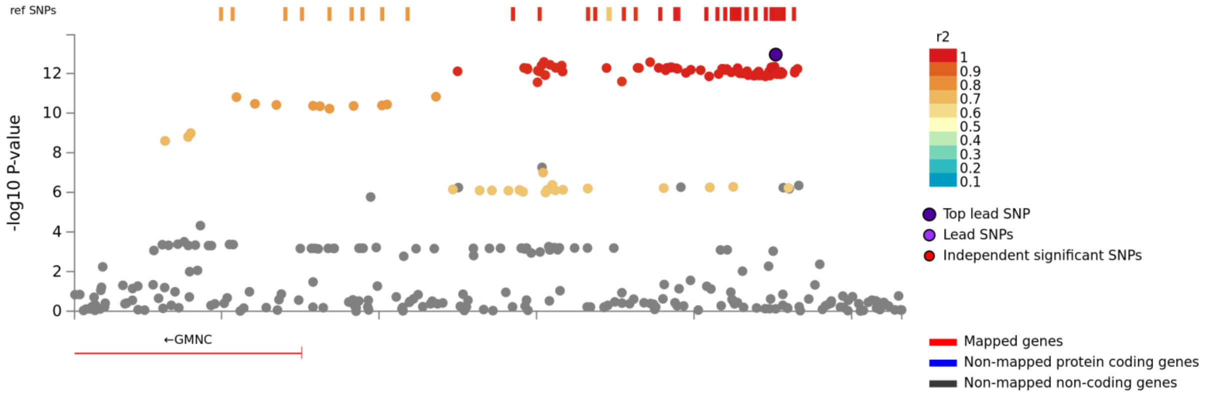

e Intralaminar nuclei-associated locus at chromosome 1

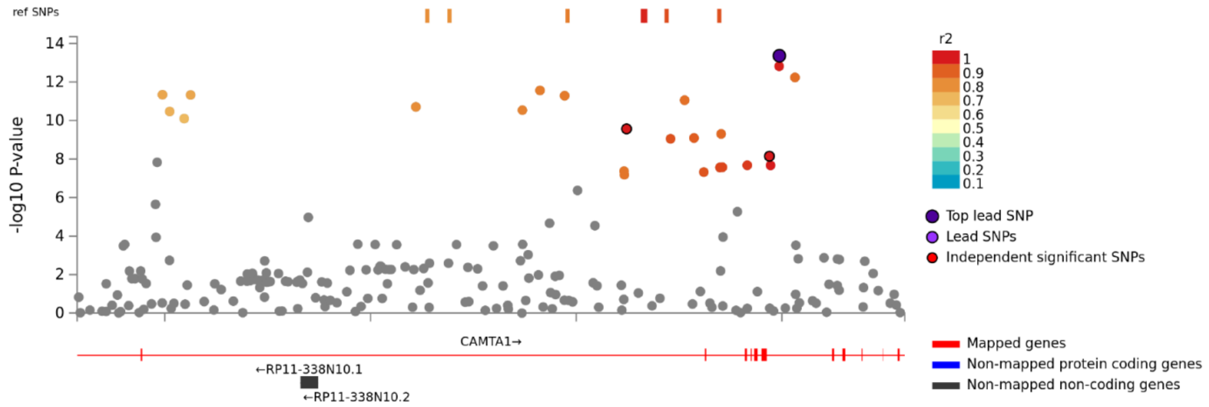

f Medial nuclei-associated locus at chromosome 3

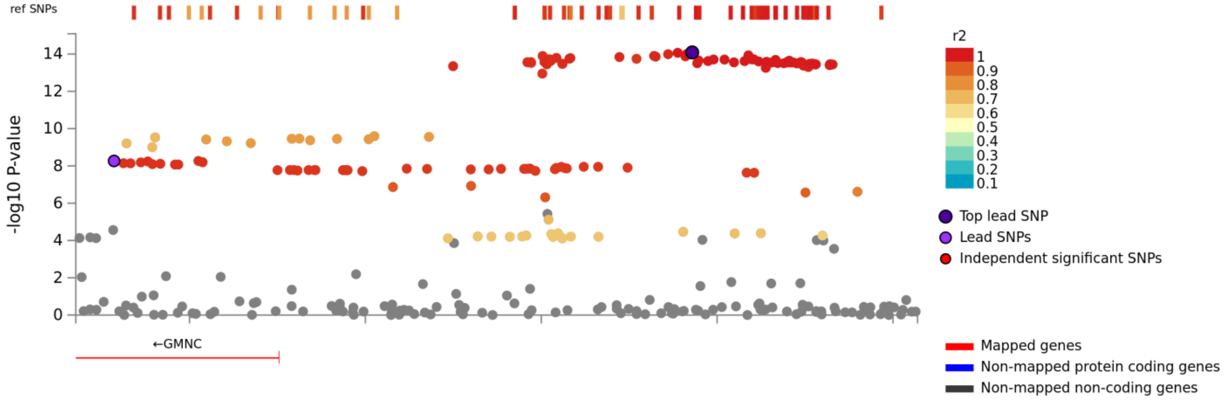

g Posterior nuclei-associated locus at chromosome 3

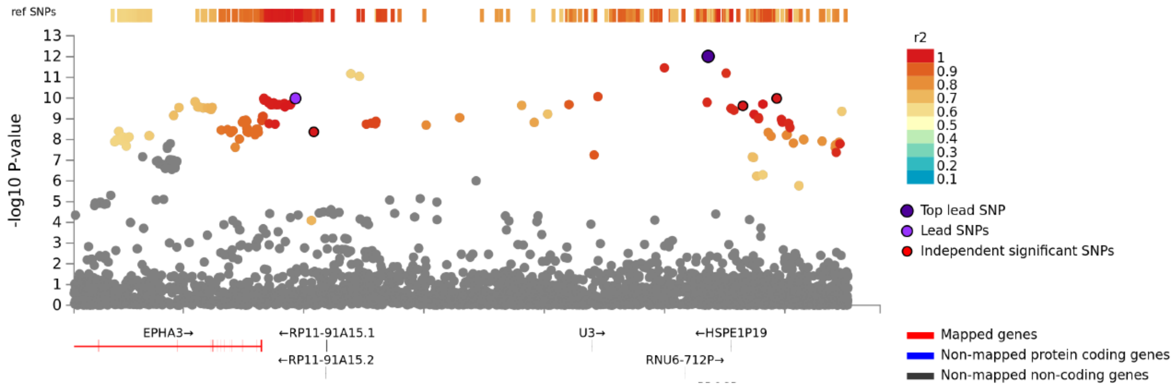

Supplementary Fig. 1 | Regional plots for the most significant genetic locus from the GWAS for volumes of the whole thalamus at chromosome 7 (**a**), anterior nuclei at chromosome 4 (**b**), lateral nuclei at chromosome 11 (**c**), ventral nuclei at chromosome 3 (**d**), intralaminar nuclei at chromosome 1 (**e**), medial nuclei at chromosome 3 (**f**), and posterior nuclei at chromosome 3 (**g**). The thalamic nuclei GWAS also accounted for whole thalamus volume. GWAS; genome-wide association studies. SNP; single-nucleotide polymorphism. Additional details can also be found at [https://fuma.ctglab.nl/browse] (FUMA ID 135-141).

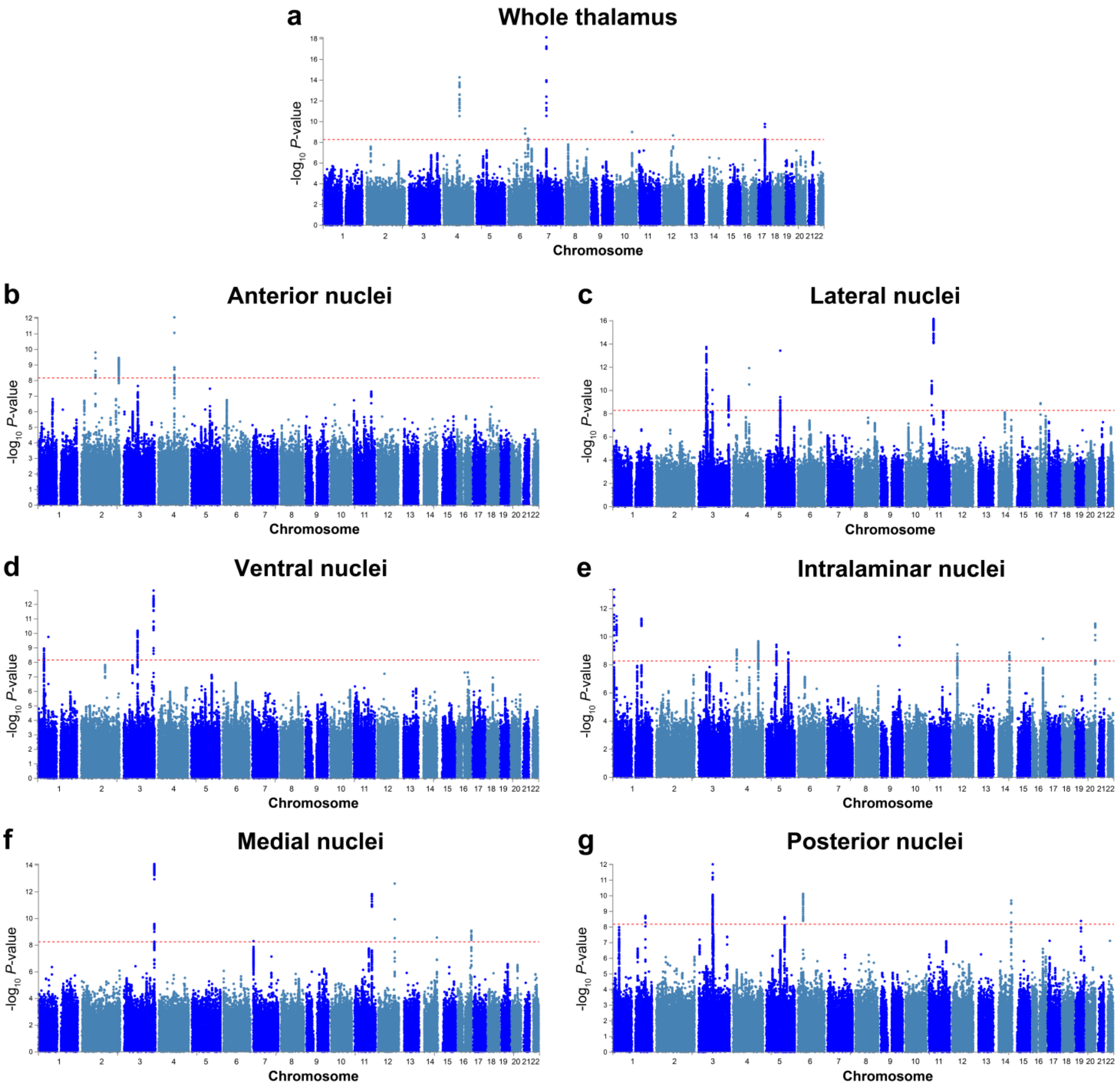

Supplementary Fig. 2 | Manhattan plots for GWAS of whole thalamus and six nuclei group volumes of the discovery sample. Seven genetic loci were associated with whole thalamus volume (**a**) and 3 loci with anterior (**b**), 11 loci with lateral (**c**), and 5 loci with ventral nuclei volumes (**d**). Twelve loci were associated with volume of the intralaminar nuclei (**e**), 6 loci with medial nuclei volume (**f**), and 6 loci with posterior nuclei volume (**g**). The analyses of the nuclei group volumes accounted for whole thalamus volume. The red horizontal lines indicate genome-wide significance after Bonferroni-correcting for analysis of seven volumes (two-sided $P < 7e\text{-}9$).

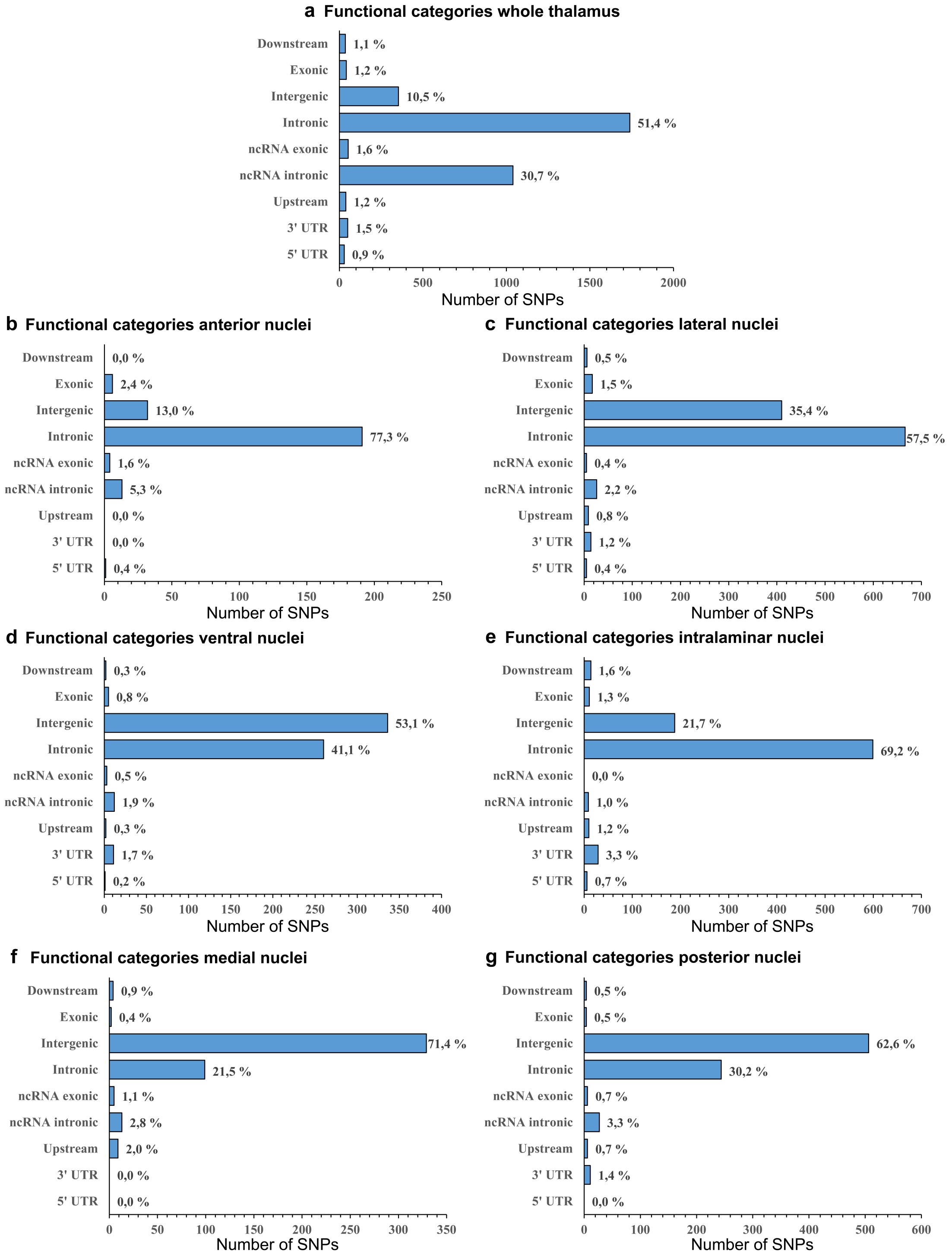

Supplementary Fig. 3 | Functional single-nucleotide polymorphisms (SNPs) categories from the genome-wide association studies for volumes of the whole thalamus (**a**), anterior nuclei (**b**), lateral nuclei (**c**), ventral nuclei (**d**), intralaminar nuclei (**e**), medial nuclei (**f**), and posterior nuclei (**g**). The figures show the distribution of single-nucleotide polymorphism across nine functional categories in percent, as indicated next to each bar, and in absolute numbers, as indicated by the x axes. ncRNA; non-coding ribonucleic acid. 5' UTR; five prime untranslated region. 3' UTR; three prime untranslated region.

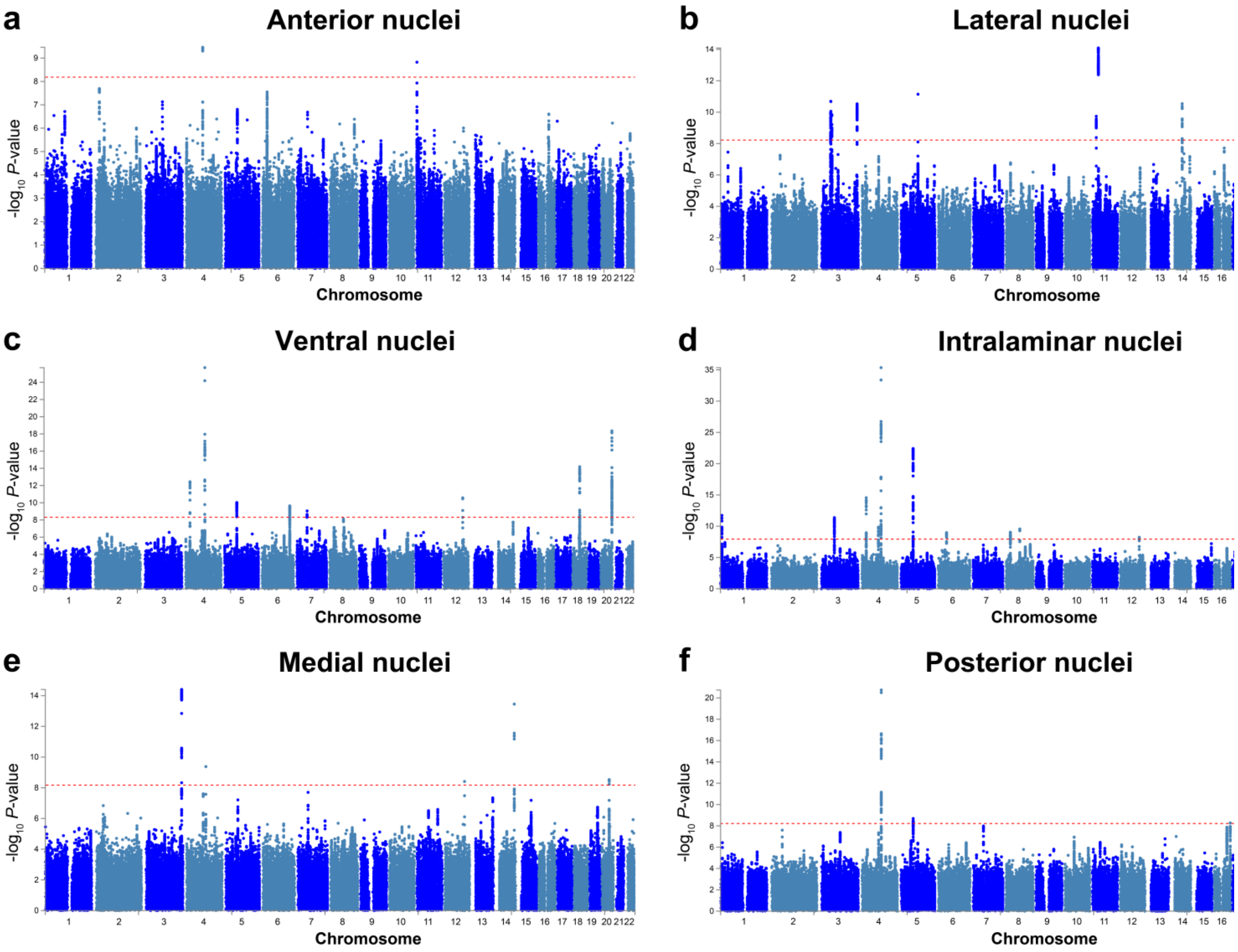

Supplementary Fig. 4 | Manhattan plots for GWAS of the six thalamic group volumes of the discovery sample when not accounting for whole thalamus volume. The GWAS for thalamic nuclei volumes were run also run without accounting for whole thalamus volume. Two genetic loci were associated with anterior (**a**), 7 loci with lateral (**b**), and 8 loci with ventral nuclei volumes (**c**). Eleven loci were associated with volume of the intralaminar nuclei (**d**), 5 loci with medial nuclei volume (**e**), and 3 loci with posterior nuclei volume (**f**). The red horizontal lines indicate genome-wide significance when the employing the same significance threshold as used for the analyses of the thalamic nuclei volumes when accounting for whole thalamus (two-sided $P < 7e\text{-}9$).

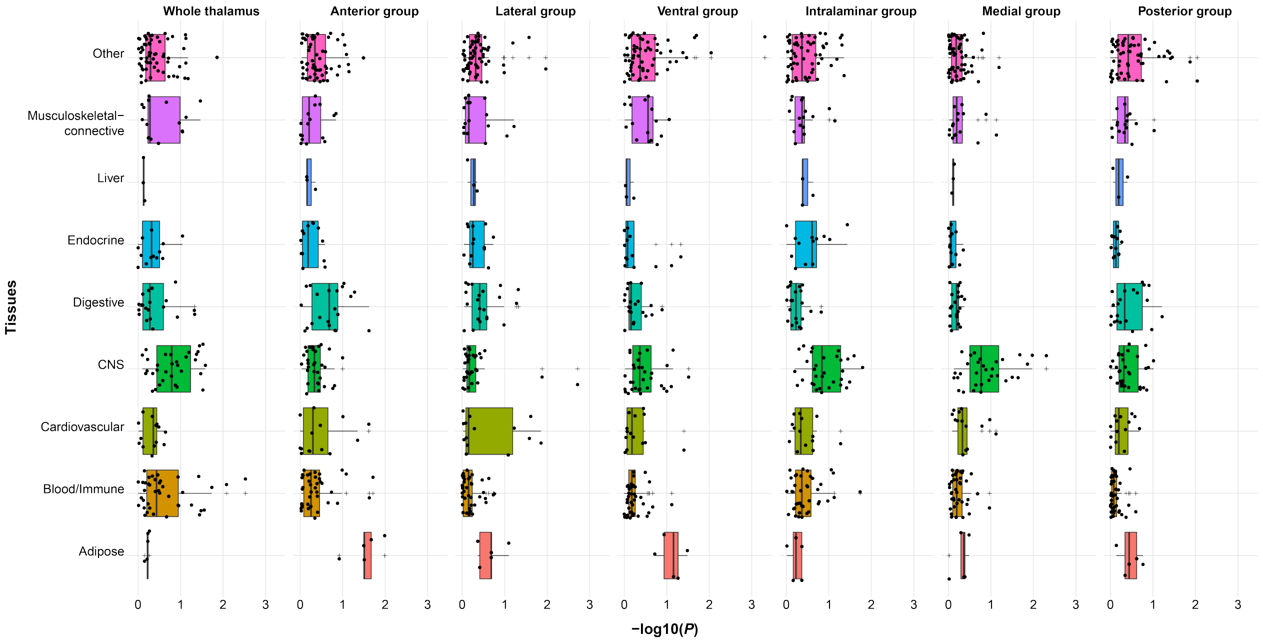

Supplementary Fig. 5 | Tissue and cell type specificity of the thalamic GWAS findings. We used linkage disequilibrium score regression applied to specifically expressed genes (LDSC-SEG) to assess the tissue and cell type specificity of the GWAS findings. Following the analyses of Finucane *et al.* (*Nature Genetics* 2018), we classified 205 tissues and cell types into nine categories for visualization, i.e., "Other", "Musculoskeletal connective", "Liver", "Endocrine", "Digestive", "CNS", "Cardiovascular", "Blood/Immune", and "Adipose". We found that volumes of the whole thalamus and intralaminar and medial nuclei groups were enriched for "CNS" tissues and cell types, yet none of these associations was significant at a Bonferroni-corrected significance threshold of P = 3.5e-5 (0.05/205 tissues and cell types/7 thalamic GWAS). The centre line indicates the median, whereas the box limits indicate the upper (75th percentile) and lower (25th percentile) quartiles. Whiskers indicate minimum and maximum data values.

a Loci for whole thalamus at chromosome 17

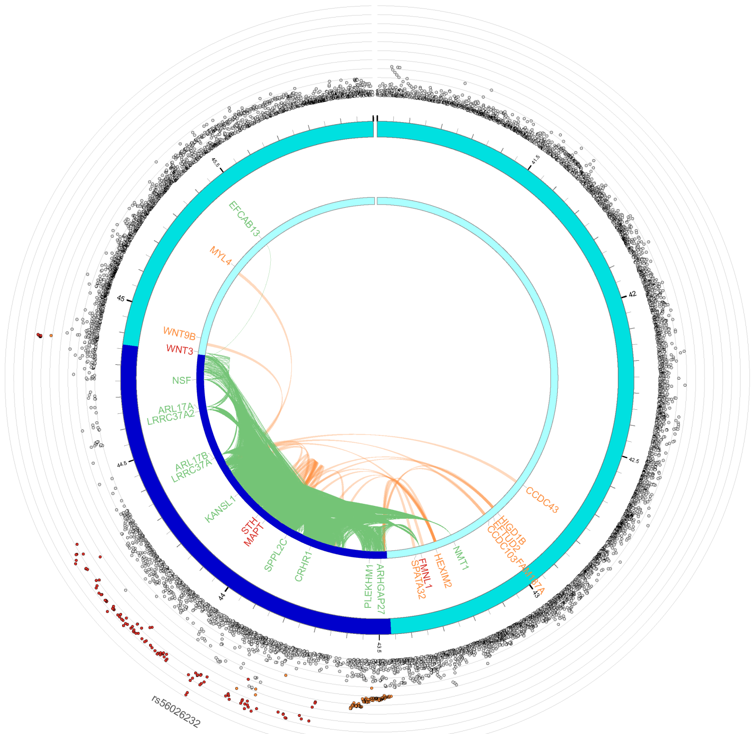

b Loci for anterior nuclei at chromosome 4

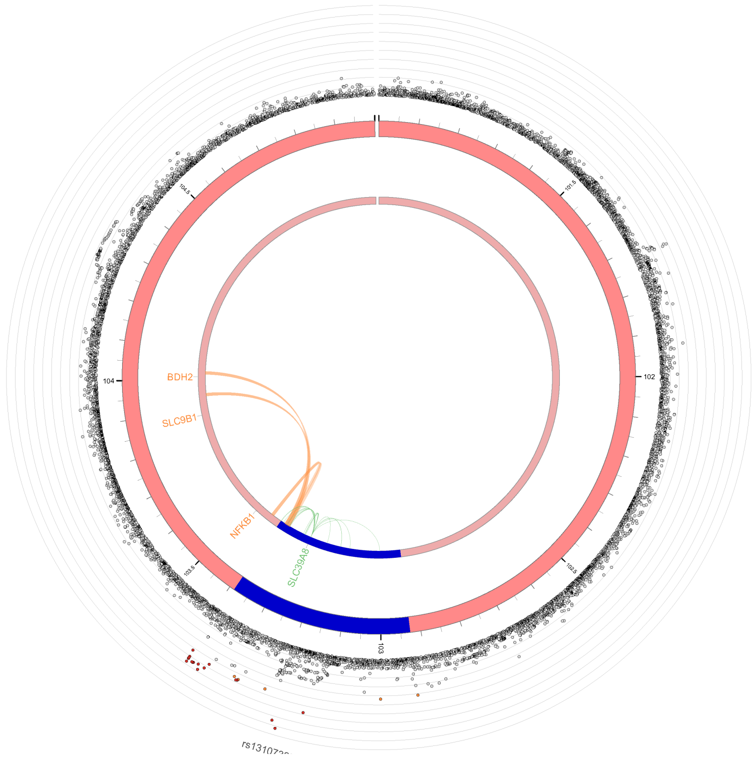

c **Loci for lateral nuclei at chromosome 3**

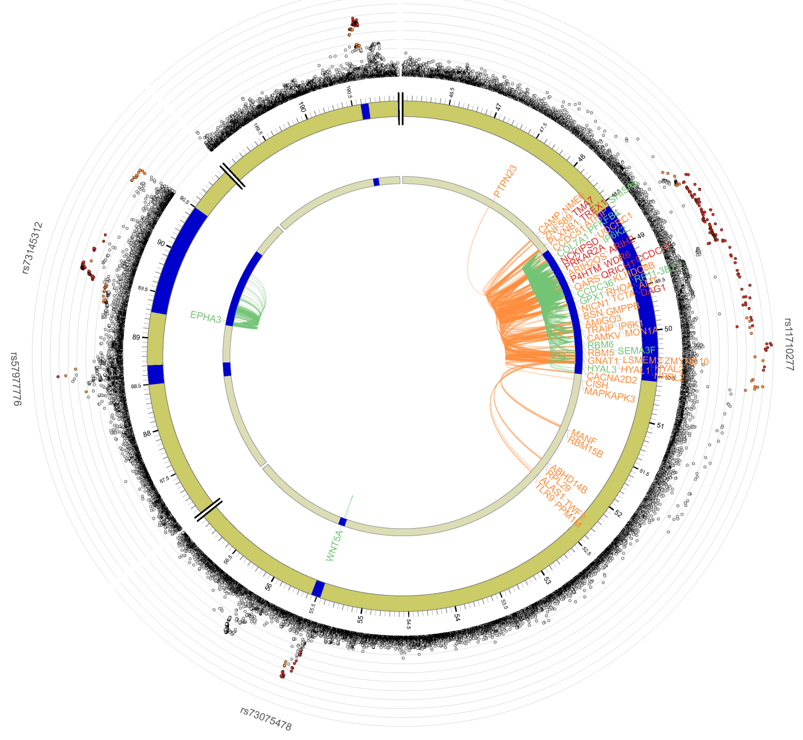

d **Loci for ventral nuclei at chromosome 1**

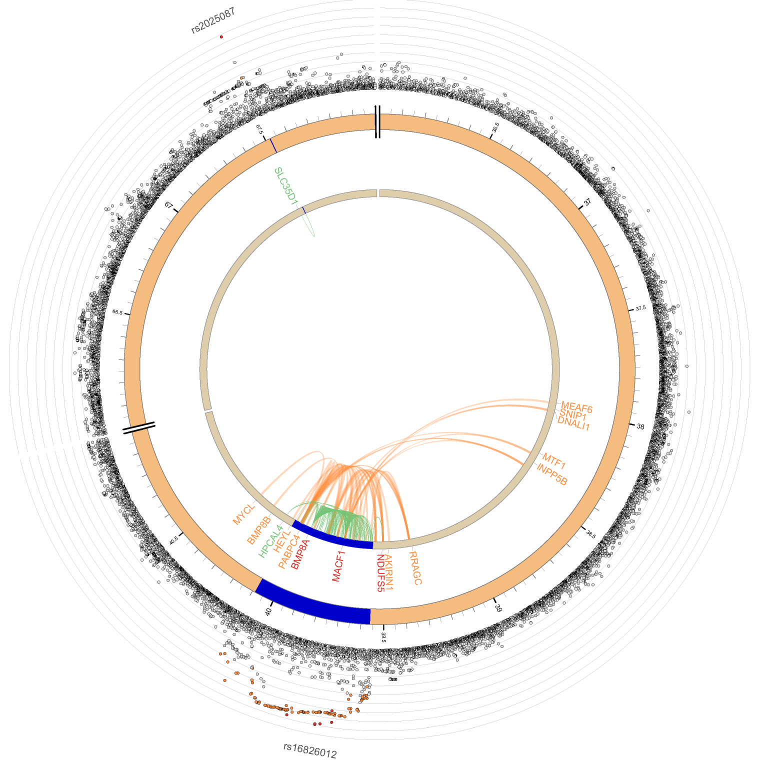

27

e Loci for intralaminar nuclei at chromosome 5

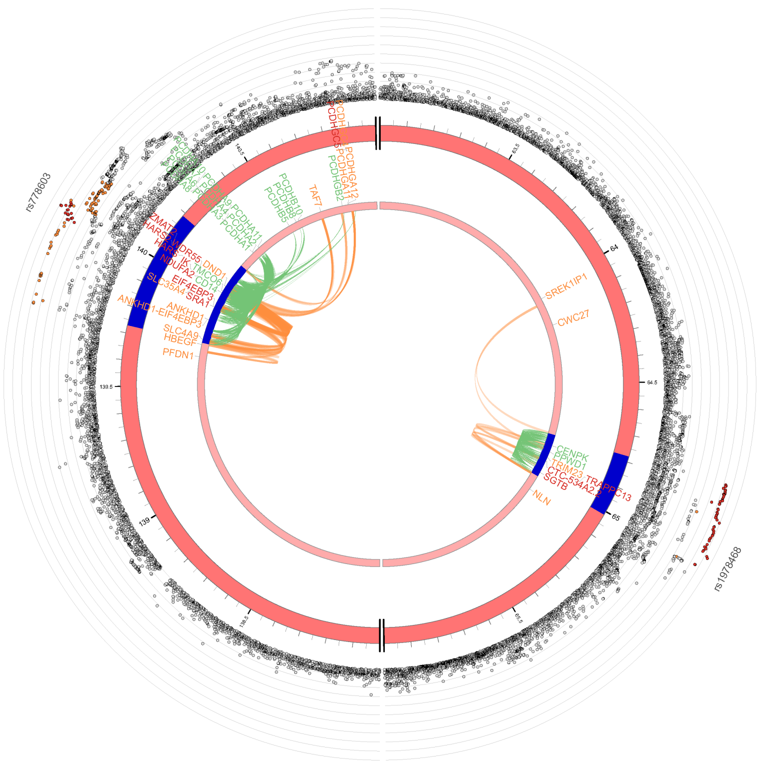

f Loci for medial nuclei at chromosome 7

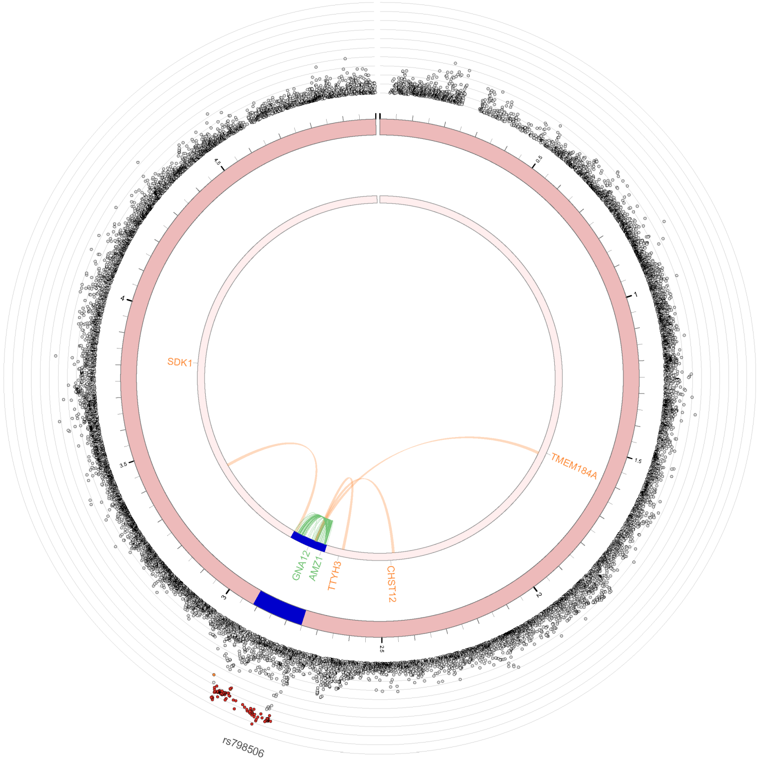

g **Loci for posterior nuclei at chromosome 19**

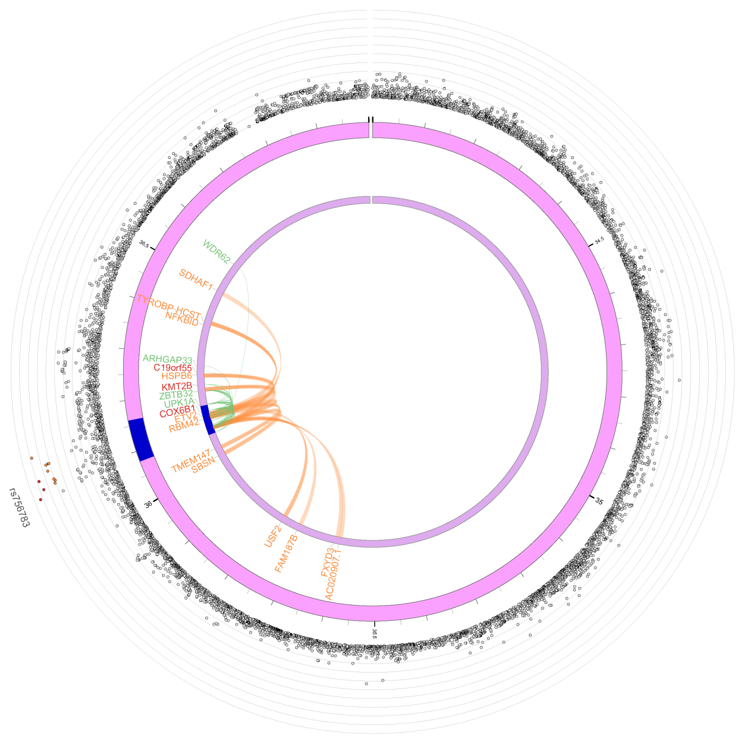

Supplementary Fig. 6 | Examples of Circos plots for mapped genes for the whole thalamus at chromosome 17 (**a**), anterior nuclei at chromosome 4 (**b**), lateral nuclei at chromosome 3 (**c**), ventral nuclei at chromosome 1 (**d**), intralaminar nuclei at chromosome 5 (**e**), medial nuclei at chromosome 7 (**f**), and posterior nuclei at chromosome 19 (**g**). The plots illustrate mapped genes of loci with $P < 7e\text{-}9$ from the GWAS of thalamus volumes (blue regions). Genes were associated with the loci by eQTL mapping (green lines) and chromatin interactions (orange lines). Green font indicates genes implicated by eQTLs, orange font indicates genes mapped by chromatin interactions, and genes implicated by both strategies are in red font. The outer layers show the Manhattan plots of single nucleotide polymorphisms from the GWAS. The statistical analyses performed for the gene mapping in Functional Mapping and Annotation of GWAS (FUMA) are described in detail on https://fuma.ctglab.nl/tutorial and the parameters employed in the present study and additional results can be found at https://fuma.ctglab.nl/browse/; ID 135-141. GWAS; genome-wide association studies.

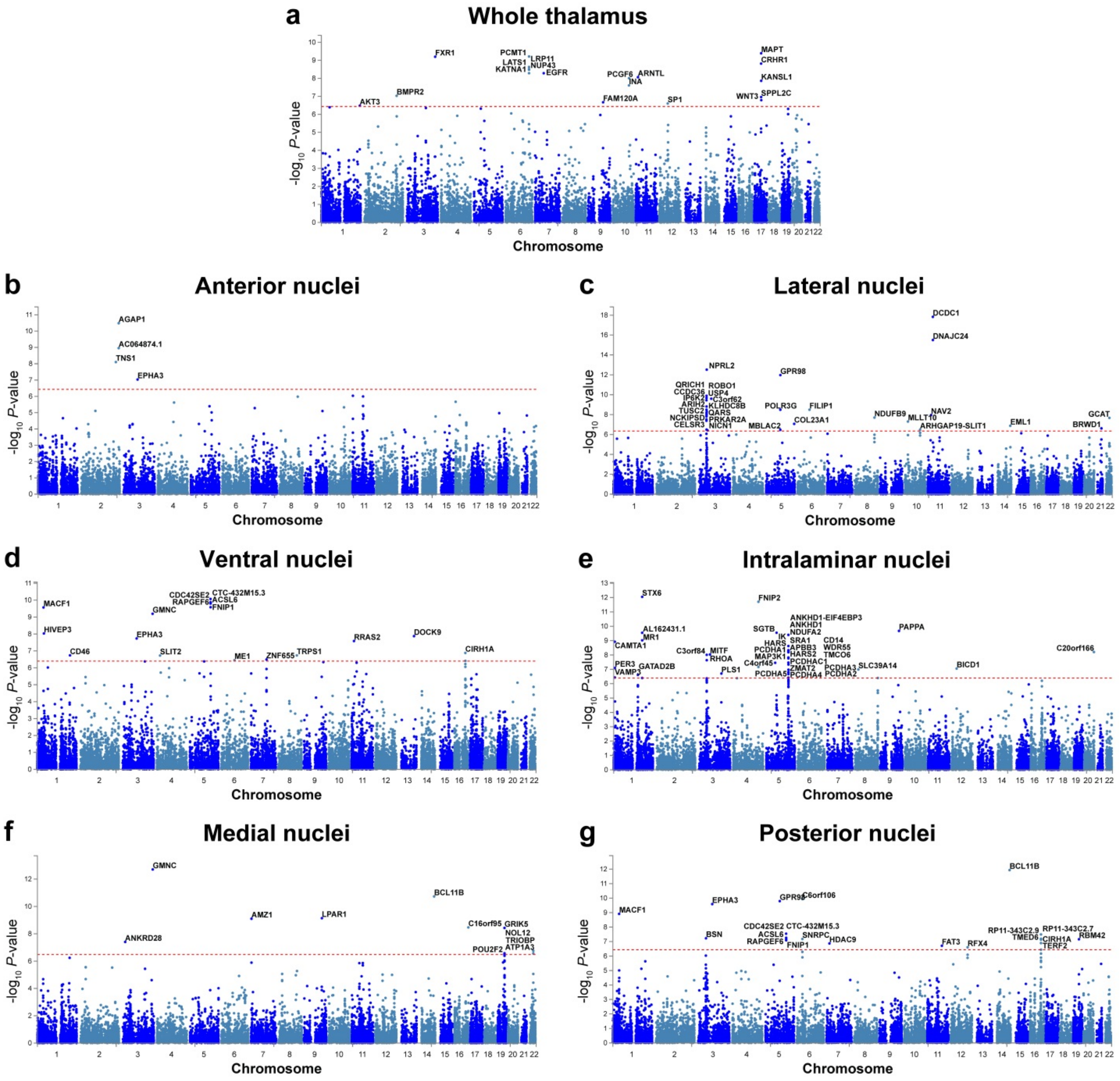

Supplementary Fig. 7 | Manhattan plots from the genome-wide gene-based association analyses for volumes of the whole thalamus (a) and volumes of the anterior (b), lateral (c), ventral (d), intralaminar (e), medial (f), and posterior (g) nuclei groups. Nineteen genes were associated with whole thalamus, 4, 29, and 17 genes were associated with volumes of the anterior, lateral, and ventral nuclei, and 37, 11, and 21 genes were associated with intralaminar, medial, and posterior nuclei volumes, respectively. Nineteen of the genes were only associated with whole thalamus volume, whereas 3, 28, 8, 37, 9, and 11 genes were only significant for volumes of the anterior, lateral, ventral, intralaminar, medial, and posterior groups. The most strongly associated gene for each volume identified by the GWGAS was MAPT (two-sided P = 4.1e-10), AGAP1 (two-sided P = 3.3e-11), DCDC1 (two-sided P = 1.5e-18), and CDC42SE2 (two-sided P = 8.5e-11) for the whole thalamus,

anterior, lateral, and ventral nuclei, respectively, and STX6 (two-sided P = 9.0e-13), GMNC (two-sided P = 2.0e-13), and BCL11B (two-sided P = 1.2e-12) for the intralaminar, medial, and posterior nuclei. The red horizontal lines indicate genome-wide significance threshold of two-sided P < 3.9e-7, i.e., 0.05/18,447 genes/7 volumes.

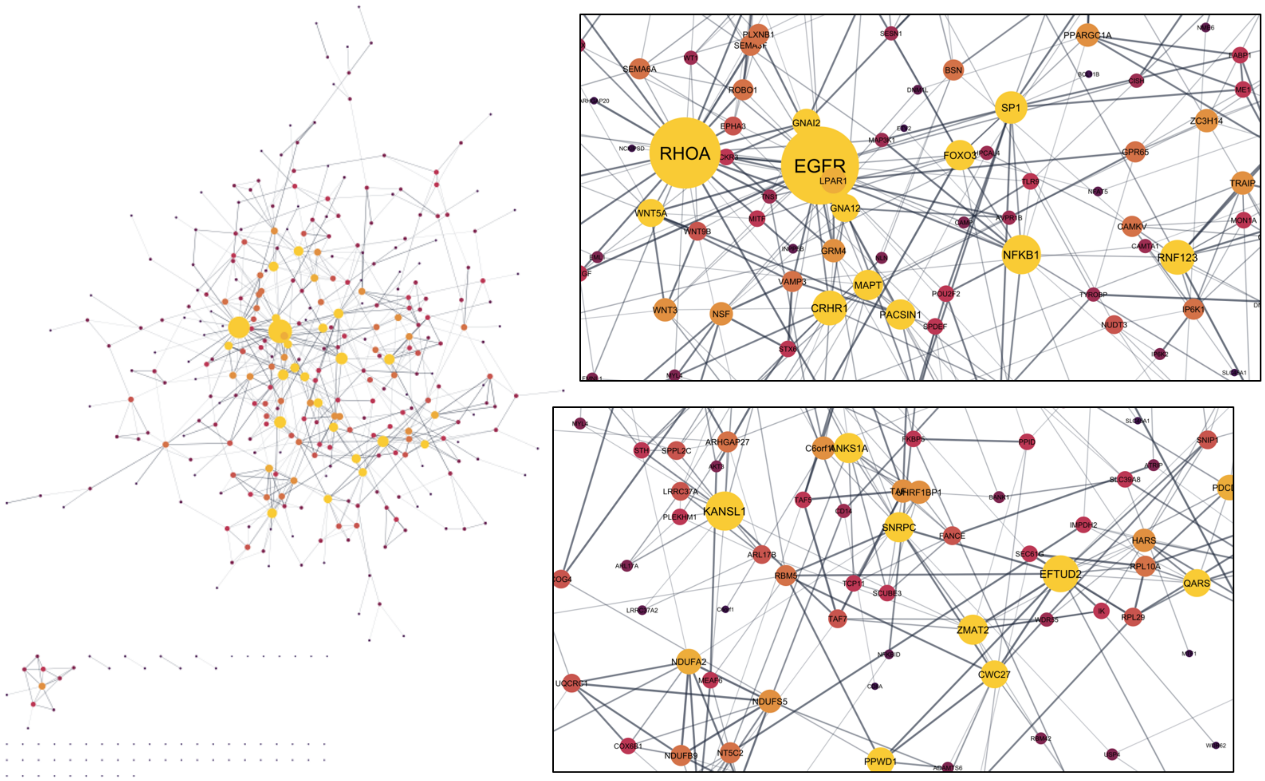

Supplementary Fig. 8 | Protein-protein interaction analysis. We conducted protein-protein interaction analysis using STRING [string-db.org] to explore the functional relationships between proteins encoded by the 391 thalamus-linked genes and exported the network to Cytoscape 3.8.2. for further analyses. We detected a network with significantly more interactions than expected by chance (protein-protein interaction enrichment: $P < 1e\text{-}16$. The most central nodes were EGFR and RHOA, followed by KANSL1, NFKB1, EFTUD2, CRHR1, and RNF123. The figure depicts the resulting network with node color, node size and font size reflecting the respective node degrees (degree d>10 in yellow). The most central nodes were EGFR (d=32) and RHOA (d=29), followed by KANSL1 (d=15), NFKB1 (d=15), EFTUD2 (d=14), CRHR1 (d=13) and RNF123 (d=13).
