# Supplementary material for: The genetic architecture of the human thalamus and its overlap with ten common brain disorders
Source: Nat Commun. 2021 May 18;12:2909. doi: 10.1038/s41467-021-23175-z (PMC8131358; doi:10.1038/s41467-021-23175-z)
Supplement: Supplementary file 3 — Description of Additional Supplementary Files [file 41467_2021_23175_MOESM3_ESM.pdf]

Description of Additional Supplementary Files

File Name: Supplementary Data 1
Description: Genetic loci associated with thalamic volumes. Genome-wide association studies (GWAS) of 30,114 genotyped healthy individuals aged 45-82 years from the UK Biobank identified seven genetic loci associated with whole thalamus volume and 3, 11, and 5 loci with volumes of the anterior, lateral, and ventral regions, and 12, 6, and 6 were associated with volumes of intralaminar, medial, and posterior regions (two-sided $P < 5e\text{-}08/7$). All GWAS accounted for age, age^2, sex, scanning site, intracranial volume, genotyping batch, and the first twenty genetic principal components to control for population stratification. In addition, the GWAS for the six thalamic regions accounted for whole thalamus volume

File Name: Supplementary Data 2
Description: Genomic inflation factor for the GWASs. The Data file shows the genomic inflation factor for the genome-wide associaton studies of whole thalamus volume and the volumes of the six thalamic nuclei groups when accounting for whole thalamus volume.

File Name: Supplementary Data 3
Description: Functional annotation of single nucleotide polymorphisms (SNPs) in linkage disequilibrium ($r2 \geq 0.6$) with one of the independent significant SNPs for whole thalamus volume. We functionally annotated all candidate single-nucleotide polymorphisms (SNPs) that were in linkage disequilibrium ($r^2 \geq 0.6$) with one of the independent significant SNPs using Functional Mapping and Annotation of GWAS (FUMA), for whole thalamus volume. FUMA is based on information from 18 biological repositories and tools and functionally annotates GWAS results. The platform prioritizes the most likely causal SNPs and genes by combining positional, eQTL, and chromatin interaction mapping. FUMA annotates significantly associated SNPs with functional categories, combined CADD scores, RegulomeDB scores, and chromatin states. All parameters used for the present study are available at https://fuma.ctglab.nl/browse/; ID 135-141.

File Name: Supplementary Data 4
Description: Functional annotation of single nucleotide polymorphisms (SNPs) in linkage disequilibrium ($r2 \geq 0.6$) with one of the independent significant SNPs for anterior nuclei volume. We functionally annotated all candidate single-nucleotide polymorphisms (SNPs) that were in linkage disequilibrium ($r^2 \geq 0.6$) with one of the independent significant SNPs using Functional Mapping and Annotation of GWAS (FUMA), for anterior nuclei volume. FUMA is based on information from 18 biological repositories and tools and functionally annotates GWAS results. The platform prioritizes the most likely causal SNPs and genes by combining positional, eQTL, and chromatin interaction mapping. FUMA annotates significantly associated SNPs with functional categories, combined CADD scores, RegulomeDB scores, and chromatin states. All parameters used for the present study are available at https://fuma.ctglab.nl/browse/; ID 135-141.

File Name: Supplementary Data 5
Description: **Functional annotation of single nucleotide polymorphisms (SNPs) in linkage disequilibrium ($r2 \geq 0.6$) with one of the independent significant SNPs for lateral nuclei volume.** We functionally annotated all candidate single-nucleotide polymorphisms (SNPs) that were in linkage disequilibrium ($r^2 \geq 0.6$) with one of the independent significant SNPs using Functional Mapping and Annotation of GWAS (FUMA), for lateral nuclei volume. FUMA is based on information from 18 biological repositories and tools and functionally annotates GWAS results. The platform prioritizes the

most likely causal SNPs and genes by combining positional, eQTL, and chromatin interaction mapping. FUMA annotates significantly associated SNPs with functional categories, combined CADD scores, RegulomeDB scores, and chromatin states. All parameters used for the present study are available at https://fuma.ctglab.nl/browse/; ID 135-141.

File Name: Supplementary Data 6
Description: Functional annotation of single nucleotide polymorphisms (SNPs) in linkage disequilibrium (r2 ≥ 0.6) with one of the independent significant SNPs for ventral nuclei volume. We functionally annotated all candidate single-nucleotide polymorphisms (SNPs) that were in linkage disequilibrium ($r^2 \geq 0.6$) with one of the independent significant SNPs using Functional Mapping and Annotation of GWAS (FUMA), for ventral nuclei volume. FUMA is based on information from 18 biological repositories and tools and functionally annotates GWAS results. The platform prioritizes the most likely causal SNPs and genes by combining positional, eQTL, and chromatin interaction mapping. FUMA annotates significantly associated SNPs with functional categories, combined CADD scores, RegulomeDB scores, and chromatin states. All parameters used for the present study are available at https://fuma.ctglab.nl/browse/; ID 135-141.

File Name: Supplementary Data 7
Description: Functional annotation of single nucleotide polymorphisms (SNPs) in linkage disequilibrium (r2 ≥ 0.6) with one of the independent significant SNPs for intralaminar nuclei volume. We functionally annotated all candidate single-nucleotide polymorphisms (SNPs) that were in linkage disequilibrium ($r^2 \geq 0.6$) with one of the independent significant SNPs using Functional Mapping and Annotation of GWAS (FUMA), for intralaminar nuclei volume. FUMA is based on information from 18 biological repositories and tools and functionally annotates GWAS results. The platform prioritizes the most likely causal SNPs and genes by combining positional, eQTL, and chromatin interaction mapping. FUMA annotates significantly associated SNPs with functional categories, combined CADD scores, RegulomeDB scores, and chromatin states. All parameters used for the present study are available at https://fuma.ctglab.nl/browse/; ID 135-141.

File Name: Supplementary Data 8
Description: Functional annotation of single nucleotide polymorphisms (SNPs) in linkage disequilibrium (r2 ≥ 0.6) with one of the independent significant SNPs for medial nuclei volume. We functionally annotated all candidate single-nucleotide polymorphisms (SNPs) that were in linkage disequilibrium ($r^2 \geq 0.6$) with one of the independent significant SNPs using Functional Mapping and Annotation of GWAS (FUMA), for medial nuclei volume. FUMA is based on information from 18 biological repositories and tools and functionally annotates GWAS results. The platform prioritizes the most likely causal SNPs and genes by combining positional, eQTL, and chromatin interaction mapping. FUMA annotates significantly associated SNPs with functional categories, combined CADD scores, RegulomeDB scores, and chromatin states. All parameters used for the present study are available at https://fuma.ctglab.nl/browse/; ID 135-141.

File Name: Supplementary Data 9
Description: Functional annotation of single nucleotide polymorphisms (SNPs) in linkage disequilibrium (r2 ≥ 0.6) with one of the independent significant SNPs for posterior nuclei volume. We functionally annotated all candidate single-nucleotide polymorphisms (SNPs) that were in linkage disequilibrium ($r^2 \geq 0.6$) with one of the independent significant SNPs using Functional Mapping and Annotation of GWAS (FUMA), for posterior nuclei volume. FUMA is based on information from 18 biological repositories and tools and functionally annotates GWAS results. The platform prioritizes the most likely causal SNPs and

genes by combining positional, eQTL, and chromatin interaction mapping. FUMA annotates significantly associated SNPs with functional categories, combined CADD scores, RegulomeDB scores, and chromatin states. All parameters used for the present study are available at https://fuma.ctglab.nl/browse/; ID 135-141.

File Name: Supplementary Data 10
Description: GWAS results in the replication sample. We conducted genome-wide association studies (GWAS) of the thalamic volumes in an independent sample of 5,173 participants from the UK Biobank. We found that 53 out of the 55 lead single nucleotide polymorphisms (SNPs) of the discovery sample had the same effect directions in the replication sample. Furthermore, 58% of the lead SNPs significant in the discovery GWAS had uncorrected two-sided P < 0.05 in the replication GWAS. Given the restricted size of the replication sample none of the variants passed the Bonferroni-corrected genome-wide threshold at 7e-09.

File Name: Supplementary Data 11
Description: Mapping of significant loci from the genome-wide association studies of thalamic volumes to genes. We used positional, expression quantitative trait loci (eQTL), and chromatin interaction mapping in the Functional Mapping and Annotation of GWAS (FUMA) platform to map the 77 independent significant singlenucleotide polymorphisms to genes. These three strategies identified 336 unique genes across the seven volumes

File Name: Supplementary Data 12
Description: Genome-wide gene-based association analyses of volumes of the whole thalamus and six thalamic regions. Genome-wide gene-based association studies (GWGAS) of 30,114 genotyped healthy individuals aged 40-70 years from the UK Biobank identified 127 unique genes across the thalamus volumes (all two-sided P = 0.05/18158 genes/7 volumes = 3.9e-7). Nineteen genes were associated with whole thalamus volume and 4, 29, 17, 37, 11 and 21 genes were associated with volumes of the anterior, lateral, ventral, intralaminar, medial, and posterior groups, respectively. Nineteen of the genes were only associated with whole thalamus volume, whereas 3, 28, 8, 37, 9, and 11 genes were only significant for volumes of the anterior, lateral, ventral, intralaminar, medial, and posterior groups. These genes are highlighted in bold below. All GWGAS accounted for age, age^2, sex, scanning site, intracranial volume, genotyping batch, and the first twenty genetic principal components to control for population stratification. In addition, the GWGAS for the anterior, lateral, ventral, intralaminar, medial, and posterior groups accounted for whole thalamus volume.

File Name: Supplementary Data 13
Description: Gene sets implicated by the significant genes. We conducted a gene-set analysis for curated gene sets and GO terms obtained from MsigDB using hypergeometric tests in Functional Mapping and Annotation of GWAS (FUMA). The tests are described in detail on https://fuma.ctglab.nl/tutorial and all parameters used for the present study are available at https://fuma.ctglab.nl/browse/; ID 135-141. This identified 4 significant gene sets for the intralaminar nuclei and 4 significant sets for the lateral nuclei, after Bonferroni correction for multiple comparisons. There were no significant Gene Ontology sets for the other volumes.

File Name: Supplementary Data 14
Description: Protein-protein interaction analysis. We conducted protein-protein interaction analysis using STRING [stringdb.org] to explore the functional relationships between proteins encoded by the 391 thalamus-linked genes and exported the network to Cytoscape 3.8.2. for further analyses. We

detected a network with significantly more interactions than expected by chance (protein-protein interaction enrichment: P < 1e-16. The most central nodes were EGFR and RHOA, followed by KANSL1, NFKB1, EFTUD2, CRHR1, and RNF123.

File Name: Supplementary Data 15
Description: Thalamocortical genetic correlations. We derived volumes of 180 cortical regions (Glasser et al. Nature 2016) and performed GWAS for each region, following the implementation performed for the thalamus volumes. Next, we assessed genetic correlation between volumes of thalamic nuclei groups and cortical regions using LD-score regression and adjusted the two-sided P-values across all performed analyses (7 thalamic volumes x 180 cortical volumes) using FDR correction in R statistics. Significant genetic correlations are shown in bold font.

File Name: Supplementary Data 16
Description: Genetic loci shared between thalamic volumes and ten psychiatric and neurological disorders. We performed conjunctional false discovery rate (FDR) analyses to detect genetic loci jointly associated with thalamic volumes and ten brain disorders. These analyses revealed shared loci across the brainstem structures and the clinical conditions. We found the largest number of loci shared between thalamic volumes and SCZ (66), PD (26), and BD (15), when applying a conjunctional FDR threshold of 0.05. For ASD, ADHD, MD, MS, GEP, FEP, and MS there were 8, 8, 17, 10, 14, 4, and 14 genetic loci jointly associated with thalamic volumes and disorders, respectively. When using a more stringent conjunctional FDR threshold of 0.01, there were pleiotropic loci associated with thalamic volumes and SCZ (17), PD (14), BD (5), ASD (2), ADHD (1), MDD (3), MS (2), and AD (2), and no shared locus for GEP or FEP. AD; Alzheimer's disease. ADHD; attention deficit hyperactivity disorder. ASD; autism spectrum disorder. BD; bipolar disorder. FEP; focal epilepsy. GEP; generalized epilepsy. MD; major depression. MS; multiple sclerosis. PD; Parkinson's disease. SCZ; schizophrenia.
