# Supplementary material for: The genetic architecture of the human thalamus and its overlap with ten common brain disorders
Source: Nat Commun. 2021 May 18;12:2909. doi: 10.1038/s41467-021-23175-z (PMC8131358; doi:10.1038/s41467-021-23175-z)
Supplement: Supplementary file 20 — Reporting Summary [file 41467_2021_23175_MOESM20_ESM.pdf]

nature research

Corresponding author(s): Torbjørn Elvsåshagen and Tobias Kaufmann

Last updated by author(s): Mar 31, 2021

Reporting Summary

Nature Research wishes to improve the reproducibility of the work that we publish. This form provides structure for consistency and transparency in reporting. For further information on Nature Research policies, see our Editorial Policies and the Editorial Policy Checklist.

Statistics

For all statistical analyses, confirm that the following items are present in the figure legend, table legend, main text, or Methods section.

n/a	Confirmed	
☐	☒	The exact sample size (*n*) for each experimental group/condition, given as a discrete number and unit of measurement
☐	☒	A statement on whether measurements were taken from distinct samples or whether the same sample was measured repeatedly
☐	☒	The statistical test(s) used AND whether they are one- or two-sided<br>*Only common tests should be described solely by name; describe more complex techniques in the Methods section.*
☐	☒	A description of all covariates tested
☐	☒	A description of any assumptions or corrections, such as tests of normality and adjustment for multiple comparisons
☐	☒	A full description of the statistical parameters including central tendency (e.g. means) or other basic estimates (e.g. regression coefficient) AND variation (e.g. standard deviation) or associated estimates of uncertainty (e.g. confidence intervals)
☐	☒	For null hypothesis testing, the test statistic (e.g. *F*, *t*, *r*) with confidence intervals, effect sizes, degrees of freedom and *P* value noted<br>*Give P values as exact values whenever suitable.*
☒	☐	For Bayesian analysis, information on the choice of priors and Markov chain Monte Carlo settings
☒	☐	For hierarchical and complex designs, identification of the appropriate level for tests and full reporting of outcomes
☐	☒	Estimates of effect sizes (e.g. Cohen's *d*, Pearson's *r*), indicating how they were calculated

Our web collection on statistics for biologists contains articles on many of the points above.

Software and code

Policy information about availability of computer code

Data collection	We included raw T1-weighted 3D brain magnetic magnetic resonance imaging and genotype data from white British participants from the UK Biobank. Data from n = 30,432 were obtained for the discovery sample and from n = 5,266 for the replication sample.
Data analysis	- The MRI data was analyzed using Freesurfer 6.0 and Bayesian thalamus segmentation, as described by Iglesias et al (Neuroimage 2018).<br>- Genome-wide association studies were run using PLINK v2.0 and the Functional Mapping and Annotation of GWAS (FUMA) platform v.1.3.5.<br>- Genome-wide gene-based association analyses (GWGAS) were run using MAGMA v1.07 in FUMA v.1.3.5.<br>- conjunctional FDR analyses were run using in-house custom software, available for download at https://github.com/precimed/pleiofdr, and MATLAB 2017a and Python 3.7.4.<br>- Statistical analyses and figures were generated using R 3.5.<br>- LD Score regression v1.0.0 (https://github.com/bulik/ldsc)<br>- LDSC-SEG (https://github.com/bulik/ldsc/)<br>- STRING (string-db.org)<br>- Cytoscape 3.8.2. (https://cytoscape.org/)<br>- ChromHMM 1.00 (http://compbio.mit.edu/ChromHMM/)

For manuscripts utilizing custom algorithms or software that are central to the research but not yet described in published literature, software must be made available to editors and reviewers. We strongly encourage code deposition in a community repository (e.g. GitHub). See the Nature Research guidelines for submitting code & software for further information.

Data

Policy information about availability of data

All manuscripts must include a data availability statement. This statement should provide the following information, where applicable:

- Accession codes, unique identifiers, or web links for publicly available datasets
- A list of figures that have associated raw data
- A description of any restrictions on data availability

Data used in this article were obtained from the UK Biobank [https://www.ukbiobank.ac.uk/], from the Psychiatric Genomics Consortium [https://www.med.unc.edu/pgc/], 23andMe [https://www.23andme.com/], the International Genomics of Alzheimer's Project [http://web.pasteur-lille.fr/en/recherche/u744/igap/igap_download.php], the International Multiple Sclerosis Genetics Consortium [http://imsgc.net/], the International Parkinson Disease Genomics Consortium [https://pdgenetics.org/], and from The International League Against Epilepsy Consortium on Complex Epilepsies [https://www.ilae.org/]. The PD GWAS partly included data from the 23andMe consortium and is available through 23andMe to qualified researchers under an agreement with 23andMe that protects the privacy of the 23andMe participants. Please visit research.23andme.com/collaborate/#publication for more information and to apply to access the data. GWAS results are also available on the FUMA website [https://fuma.ctglab.nl/browse/; ID 135-141]. The summary statistics for thalamic volumes of the present study are publicly available on GitHub [https://github.com/norment/open-science/tree/main/2021_Elvsashagen_NatComms_ThalamusGenetics].

Field-specific reporting

Please select the one below that is the best fit for your research. If you are not sure, read the appropriate sections before making your selection.

- [x] Life sciences
- [ ] Behavioural & social sciences
- [ ] Ecological, evolutionary & environmental sciences

For a reference copy of the document with all sections, see nature.com/documents/nr-reporting-summary-flat.pdf

Life sciences study design

All studies must disclose on these points even when the disclosure is negative.

Sample size	The present study includes raw T1 MRI data from n = 35,698 genotyped white British from the UK Biobank (discovery sample n = 30,432; replication sample n = 5,266). No prior GWAS of individual thalamus nuclei has been conducted and no formal power analyses or sample size estimations were conducted. We included all imaging-genetics data that was available to us in the UK Biobank.
Data exclusions	We manually assessed the thalamus segmentations in all MRI data sets by visually inspecting axial view figures of the delineations for each participant. This procedure excluded 318 data sets from the discovery sample (due to tumors and other lesions (6%), cysts (12%), ventricle abnormalities (18%), segmentation errors (14%), and insufficient data quality (50%)) and 93 data sets from the replication sample (due to tumors and other lesions (4%), cysts (13%), ventricle abnormalities (13%), segmentation errors (9%), and insufficient data quality (61%)). Thus, the final sizes of the discovery and replication GWAS samples were n = 30,114 and n = 5,173, respectively. These criteria for exclusion were pre-established.
Replication	The thalamus-associated lead SNPs of the discovery sample with P < 7e-9 were also evaluated in the GWAS replication sample. 96% of these lead SNPs had the same effect direction in the replication (sign test; P = 8.6e-14). Moreover, 58% of the discovery lead SNPs had uncorrected P < 0.05 in the replication Due to the modest size of the replication sample and the limited statistical power, there were no attempts at replicating the post-GWAS findings of the present study. These include gene mapping and gene sets analyses, and analyses of genetic overlap between brainstem volumes and common brain disorders.
Randomization	No randomization was conducted since the study did not include a design e.g., a placebo-controlled treatment trial, where randomization was relevant. The analyses were run on all available data.
Blinding	No blinding was conducted since the study did not include a design e.g., a placebo-controlled treatment trial, where blinding was relevant.

Reporting for specific materials, systems and methods

We require information from authors about some types of materials, experimental systems and methods used in many studies. Here, indicate whether each material, system or method listed is relevant to your study. If you are not sure if a list item applies to your research, read the appropriate section before selecting a response.

Materials & experimental systems

n/a	Involved in the study
☒	☐ Antibodies
☒	☐ Eukaryotic cell lines
☒	☐ Palaeontology and archaeology
☒	☐ Animals and other organisms
☐	☒ Human research participants
☒	☐ Clinical data
☒	☐ Dual use research of concern

Methods

n/a	Involved in the study
☒	☐ ChIP-seq
☒	☐ Flow cytometry
☐	☒ MRI-based neuroimaging

Human research participants

Policy information about studies involving human research participants

Population characteristics	The present study includes n = 35,698 genotyped white British from the UK Biobank. After QC procedures, the final sizes of the discovery (52% females; age range 45-82 years) and replication (51% females; age range 46-81 years) GWAS samples were n = 30,114 and n = 5,173, respectively.
Recruitment	UK Biobank conducted its recruitment phase in 2007-2010 in which 500,000 participants gave their consent. Details concerning recruitment procedures of the UK Biobank can be found at https://www.ukbiobank.ac.uk/key-documents/. We are not aware of recruitment biases that are likely to have a major impact on the results obtained in the current study.
Ethics oversight	The National Health Service National Research Ethics Service (ref. 11/112 NW/0382) has approved the UK Biobank. The projects from which summary statistics of GWAS were used for genetic overlap analysis were each approved by the local ethics committees, and informed consent was obtained from all participants, as cited in the manuscript file. The Norwegian Regional Committees for Medical and Health Research Ethics (REC South East) evaluated our pipelines that use summary statistics from published works for genetic analysis as performed in the current study and found that no additional institutional approval is needed.

Note that full information on the approval of the study protocol must also be provided in the manuscript.

Magnetic resonance imaging

Experimental design

Design type	Structural MRI
Design specifications	The MRI data were used for GWAS, genetic correlation, and genetic overlap analyses.
Behavioral performance measures	Only structural MRI was used in the present study and no behavioral performance was measured.

Acquisition

Imaging type(s)	Structural MRI, T1-weighted
Field strength	3T
Sequence & imaging parameters	TR=2000ms, TE=2.01ms, FA=8° (3 identical scanning sites), employing a Siemens 3T Skyra.
Area of acquisition	Whole brain

Diffusion MRI ☐ Used ☒ Not used

Preprocessing

Preprocessing software	The MRI data was processed using recon -all in Freesurfer 5.3 and the thalamus and its nuclei were then delineated using Bayesian thalamus segmentation in Freesurfer 6.0, as described by Iglesias et al (Neuroimage 2018).
Normalization	Standard procedures employed in Freesurfer (recon -all) were employed.
Normalization template	fsaverage
Noise and artifact removal	We used standard pipelines for anatomical data (Freesurfer recon -all).
Volume censoring	We did not employ volume censoring.

Statistical modeling & inference

Model type and settings	We conducted GWAS with PLINK on whole thalamus and the six thalamic nuclei volumes (summed for left and right thalamus) accounting for age, age-orthogonalized age squared, sex, scanning site, intracranial volume, and the first twenty genetic principal components. The thalamic nuclei GWAS were run with and without covarying for whole thalamus volume and the significance threshold was Bonferroni-corrected for analyses of seven volumes (P=5e-08/7=7e-09.). We used linkage disequilibrium score regression applied to specifically expressed genes (LDSC-SEG) to assess the tissue and cell type specificity of the GWAS findings (https://github.com/bulik/ldsc/wiki/Cell-type-specific-analyses). Here, we used a Bonferroni-corrected significance threshold of P = 0.05/205 tissues and cell types/7 thalamic GWAS = 3.5e-5. We conducted protein-protein interaction analysis using STRING [string-db.org] to explore the functional relationships between proteins encoded thalamus-linked genes and exported the network to Cytoscape 3.8.2. [https://cytoscape.org/] for further analyses. We assessed protein-protein interaction enrichment and node degrees of the resulting network. We assessed genetic correlation between thalamic nuclei groups and cortical regions using LD-score regression and adjusted the P-values across all performed analyses (7 thalamic volumes x 180 cortical volumes) using FDR correction. We conducted genetic overlap analyses between thalamic volumes and then brain disorders using conjunctional FDR analyses; these analyses were run with FDR-thresholds of both 0.05 and 0.01.
Effect(s) tested	We assessed the effects of SNPs on whole thalamus and the six thalamic nuclei volumes, the genetic correletations between the thalamic and cortical volumes, and the genetic overlap between thalamic volumes and ten brain disorders.

Specify type of analysis: ☐ Whole brain ☒ ROI-based ☐ Both

Anatomical location(s)	The present study analyzed volumes of the whole thalamus and and six thalamic nuclei groups, i.e., anterior, lateral, ventral, intralaminar, medial, and posterior nuclei groups. Volumes for left and right thalamus were summed and these were used in the analyses.
Statistic type for inference (See Eklund et al. 2016)	Standard univariate GWAS and replication in independent data.
Correction	The GWAS of thalamic volumes and the post-GWAS analyses were run with Bonferroni-corrections for analyses of seven volumes. Conjunctional FDR-analyses were adjusted for multiple testing using FDR.

Models & analysis

n/a	Involved in the study
☒	☐ Functional and/or effective connectivity
☒	☐ Graph analysis
☒	☐ Multivariate modeling or predictive analysis
